# Comparative Analysis of Heavy Metal Content in Impacted Third Molars from Industrial and Non-Industrial Areas and Its Effect on the Isolation, Culture, and Proliferation of Dental Stem Cells (DSCs)

**DOI:** 10.3390/jcm13185465

**Published:** 2024-09-14

**Authors:** Benita Wiatrak, Sadri Rayad, Tomasz Gębarowski, Jakub Hadzik, Marzena Styczyńska, Tomasz Gedrange, Maciej Dobrzyński, Ewa Barg, Marzena Dominiak

**Affiliations:** 1 Department of Pharmacology, Faculty of Medicine, Wroclaw Medical University, Mikulicza-Radeckiego 2, 50-345 Wroclaw, Poland; benita.wiatrak@umw.edu.pl; 2 Academic Dental Polyclinic of Dental Center, Technology Transfer Ltd., Krakowska 26, 50-425 Wroclaw, Poland; sadri.rayad@gmail.com; 3 Department of Biostructure and Animal Physiology, The Wroclaw University of Environmental and Life Sciences, Kożuchowska 1/3, 51-631 Wroclaw, Poland; 4 Department of Dental Surgery, Wroclaw Medical University, Krakowska 26, 50-425 Wroclaw, Poland; jakub.hadzik@umw.edu.pl (J.H.); tomasz.gedrange@umw.edu.pl (T.G.); marzena.dominiak@umw.edu.pl (M.D.); 5 Department of Human Nutrition, Wroclaw University of Environmental and Life Sciences, Chelmonskiego 37/41, 51-630 Wroclaw, Poland; marzena.styczynska@upwr.edu.pl; 6 Department of Pediatric Dentistry and Preclinical Dentistry, Wroclaw Medical University, Krakowska 26, 50-425 Wroclaw, Poland; maciej.dobrzynski@umw.edu.pl; 7 Department of Basic Medical Sciences, Wroclaw Medical University, Borowska 211A, 50-556 Wroclaw, Poland; ewa.barg@umw.edu.pl

**Keywords:** regenerative medicine, DPSCs, tissue regeneration, oral medicine

## Abstract

**Background**: This study investigates the impact of environmental pollution on the quality and viability of dental stem cells (DSCs) from impacted third molars. By comparing DSCs from patients in industrial areas with high air pollution and those from non-industrial regions, the research assesses the adverse effects of heavy metals on stem cell proliferation. **Methods**: Impacted lower third molars were collected from 28 patients—10 from industrial and 18 from non-industrial areas. Patients were divided into two age groups: 18–27 years and 28–38 years old. Dental pulp was extracted under sterile conditions, and DSCs were isolated and cultured. Heavy metal concentrations in dental tissues were measured using atomic absorption/emission spectrometry. **Results**: The study found significantly higher concentrations of copper and lead in the dental tissues of patients in industrial areas. Cell viability was lower in samples from these areas, with a statistically significant difference in average doubling time and the number of cells obtained after the first passage. There was no significant impact of gender on heavy metal content, except for higher iron levels in men. **Conclusions**: Exposure to industrial pollutants negatively affects the viability and proliferation of DSCs, but there are no differences in differentiation in the osteogenic medium regarding cell mineralization. These studies highlight the importance of environmental factors for oral health, suggesting that residents of polluted areas may face greater difficulties in dental and regenerative treatments. Further research is needed to develop strategies to mitigate the effects and improve clinical outcomes for affected populations.

## 1. Introduction

Medicine and modern dentistry increasingly utilize the potential of transplantation. Unfortunately, not every case yields a satisfactory clinical result [1,2,3]. One common approach is bone transplantation, using material from the patient (autogenous), another donor (allogeneic), or a synthetic product (xenogeneic). While autologous bone grafting remains the “gold standard”, harvesting from sites like the hip or rib is invasive and painful, leading to the use of allogeneic or xenogeneic methods, which carry different risks [1,2].

Allogeneic materials are purified using various protocols, such as heat treatment or gamma radiation, removing over 95% of leukocytes and plasma components. Though traditionally considered low risk for alloimmunization, bone grafts can trigger immune responses, which may impact treatment outcomes [4,5,6,7].

Bone grafts are often necessary in maxillofacial surgery and orthodontic treatments, where bone loss is common, even in younger patients. The goal of using stem cells is rapid bone regeneration, restoring its shape, function, and blood supply, making it an excellent option for craniofacial defects or bone atrophy caused by trauma, disease, or cancer [8,9,10].

Stem cells from bone marrow (BMSCs) were first used in medicine, but obtaining them is invasive and yields fewer cells than from dental pulp. Adipose tissue is another source, but isolating stem cells requires large volumes of fat and poses risks like brain or heart embolism [11,12,13]. In contrast, dental pulp stem cells (DSCs) offer a safer and more efficient option for obtaining mesenchymal stem cells (MSCs) [14].

The dental pulp contains odontoblasts, fibroblasts, and DSCs, including dental pulp stem cells (DPSCs), stem cells from human exfoliated deciduous teeth (SHEDs), and periodontal ligament stem cells (PDLSCs). These cells exhibit multipotent characteristics, making them valuable for regenerative treatments. Extracted teeth, often discarded, can be a rich source of DPSCs [15].

Environmental factors, particularly heavy metals, can significantly affect the quality of DSCs. Studies show that metals like cadmium, lead, and mercury accumulate in dental tissues, posing health risks. Teeth can serve as biomarkers for environmental and occupational exposure to these metals [16].

This study aimed to conduct a detailed assessment of the quality of cells obtained from patients who required the extraction of third molars. The study aimed to compare the quality of cells from patients living in industrial areas, characterized by high levels of air pollution, with the quality of cells from patients in areas with lower pollution levels. The analysis aimed to identify potential differences in cell quality that may be associated with exposure to environmental pollutants and to assess the impact of these pollutants on the cellular health of patients. The results of the study may provide valuable information on the impact of the environment on oral health and the overall health condition of patients.

## 2. Materials and Methods

Before conducting the study, approval was obtained from the Bioethics Committee of Wroclaw Medical University, Wrocław, Poland (approval number: KB-246/2019 and KB-34/2020).

### 2.1. Materials

The material consisted of impacted lower third molars collected from 28 patients: 10 residents of the Glogow-Legnica district (industrial) and 18 residents of Wroclaw (non-industrial city), who underwent third molars extraction surgery [17]. Patients of both sexes were divided into two groups: young (18–27 years old) and mature (28–38 years old). During the study, data were collected regarding gender, age, place of permanent residence, lack of occupational exposure to toxic metals, lack of smoking habits, and the use of dietary supplements. The assignment of patients to industrial or non-industrial area groups was based on self-reported place of residence, without specific inquiries into dietary habits, smoking, or occupational exposure to heavy metals. This lack of control over additional influencing factors presents a limitation of the study, as these factors could potentially affect heavy metal accumulation and DSC characteristics. The sample selection was not randomized but aimed to include a balanced number of patients from industrial and non-industrial areas, representing typical environmental exposure scenarios in Lower Silesia. Randomization was not possible due to logistical limitations, but we sought to minimize biases by excluding patients with occupational exposure to toxic metals and ensuring a similar age distribution between the groups.

### 2.2. Pulp Extraction

Before the entire procedure, the patient is informed of the possible risks and complications associated with the procedure, and then the performing doctor obtains the patient’s written and informed consent to perform the extraction. The tooth extraction procedure was carried out under local anesthesia with the patient fully conscious. The procedure was carried out in accordance with the principles of current medical knowledge and good medical practice, and the technique of the procedure was adapted each time to the clinical situation. The most common procedure involves the creation of a mucosal and bone flap; after flap dehiscence, the tooth is chiseled. The extracted tooth is then secured for the next stage to obtain material.

The cutting of the tooth to obtain the pulp tissue had to take place outside the mouth under sterile conditions so as not to cause contamination with oral bacterial flora. After removal, the teeth were stored in a cool antibiotic solution (0.5 mg/mL gentamicin—Biological Industries, Beit Haemek, Israel) in a refrigerator (temperature: 6 °C). The wisdom teeth were then cut with a sterile diamond disc with water cooling with sterile physiological saline (Biological Industries, Beit Haemek, Israel). The most effective was a combination of longitudinal and transverse cuts depending on the morphology of the tooth in question, leading to a weakening of the enamel and dentine structures, while not leading to a violation of the tooth chamber. With the use of hand tools (dental levers), access to the chamber was gained, and the intact tooth pulp was immediately transferred to the antibiotic solution.

### 2.3. Isolation of DCSCs

The obtained dental pulp was placed in the transport medium at 4 °C for up to 24 h (usually 18 h). The pulp was usually collected in the afternoon, and isolation was performed the next morning. After transport to the laboratory, the pulp was rinsed using a PBS/gentamicin solution (Biological Industries, Beit Haemek, Israel). Then the isolation process was carried out. Isolation was performed by cutting with scissors in 5 mL tubes to obtain a homogeneous suspension. The pulp was then digested using collagenase II at a concentration of 4 mg/mL in Hanks’ Balanced Salt Solution (HBSS). Before digestion, the collagenase solution (Sigma-Aldrich, Darmstadt, Germany) was filtered through 0.22 µm filters (Sigma-Aldrich, Darmstadt, Germany). Digestion time was 1 h at 37 °C. After this time, the suspension was neutralized with a complete medium and centrifuged at 200 G for 5 min at 4 °C. After centrifugation, the obtained pellet was suspended in the medium, and the viability and number of obtained cells were assessed. The obtained suspension was placed in culture flasks with a surface area of 25 cm^2^ (TPP Techno Plastic Products AG, Trasadingen, Switzerland). The first culture assessment was performed after 72 h, after which the culture medium was changed. The culture was then assessed every 48–72 h, and if confluence above 80% was achieved, the passage procedure was performed by detaching the cells using the TrypLE solution (Thermo Fisher Scientific, Waltham, MA, USA). After the first passage, the cells were further cultured in flasks with a surface area of 74 cm^2^, and their further assessment was performed—growth, surface antigen expression, and differentiation into other cell types were assessed.

### 2.4. Cell Viability

**Figure 1:**
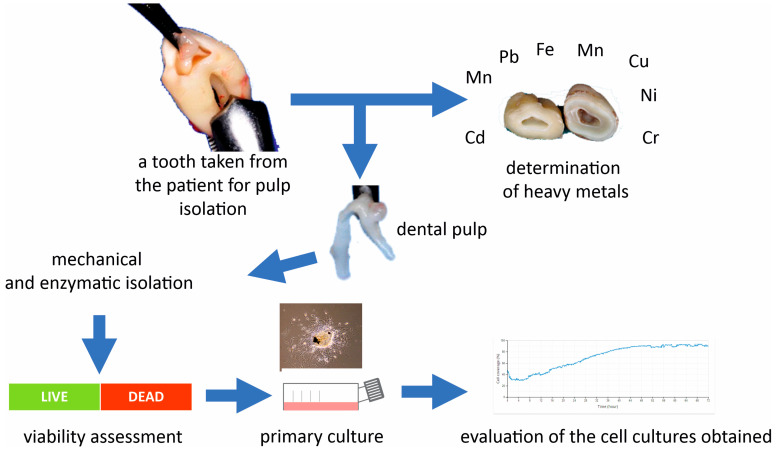
Scheme of in vitro tests performed.

The assessment of cell viability and counting was performed using a ready-made AO PI dual fluorescence kit (Sigma-Aldrich, Darmstadt, Germany) for analyzing concentration and viability according to the manufacturer’s instructions. The measurement was carried out using the Countstar reader (Alit Biotech Co., Ltd., Shanghai, China). The evaluation of the cell growth rate was conducted using the Lionheart FX (Agilent Technologies, Inc. Santa Clara, CA, USA) automated microscope (Figure 1). This microscope maintains humidity and temperature as in standard culture conditions in a CO_2_ incubator. This allowed for real-time monitoring of cell proliferation growth over 48–72 h.

### 2.5. Induction of Differentiation DSCs

When the cells reached approximately 80% confluence, the medium was replaced with osteogenic differentiation medium (0.5 mL/well; 24-well plate format). The cells were incubated with MSCgo Osteogenic XF (cat. no. 05-440-1, Biological Industries, Beit Haemek, Israel) or MSCgo Rapid Osteogenic XF (cat. no. 05-442-1, Biological Industries, Beit Haemek, Israel) for a period ranging from 10 to 21 days at 37 °C in a 5% CO_2_ atmosphere. The differentiation medium was replaced every 2 to 3 days.

The differentiation and mineralization were assessed using a 2% Alizarin Red S (ARS—Sigma-Aldrich, Darmstadt, Germany) staining solution. ARS staining was performed to qualitatively and quantitatively evaluate the extent of calcium deposition, which is indicative of osteogenic differentiation.

### 2.6. Mineralization of Research Material

The samples were “wet” mineralized in a closed microwave mineralization system MARS 6 (CEM, Matthews, NC, USA). Next, 5 cm^3^ of concentrated nitric acid (V) p.a was added to homogeneous sample (from 0.1 g to 0.5 g) in preparation vessels. Then the samples were mineralized in the microwave sample preparation system. The minerals were quantitatively transferred to 10 cm^3^ measuring vessels with redistilled water. The mineralization was carried out in accordance with the Polish Standard PN-EN 13805:2003 “Foodstuffs. Determination of trace elements. Pressure mineralization” [18].

### 2.7. Determination of Elements in the Research Material

Determination of toxic heavy metals (Mn, Cr, Ni, Cu, Fe, Pb, Cd, Zn) content in materials by atomic absorption/emission spectrometry an acetylene/air flame was carried out by atomic absorption or emission spectrometry using a SpectrAA atomic absorption spectrometer with a Varian AA240FS flame attachment (Varian Inc., Palo Alto, CA, USA).

The analyses were performed in a food testing laboratory at the Department of Food Nutrition, University of Environmental and Life Sciences in Wroclaw.

The accuracy of the method was confirmed on the basis of the Standard Reference Material, Bone Meal, NIST-1486 (National Institute of Standards and Technology, Gaithersburg, MD, USA), and the measurement uncertainty was estimated at 5%.

The elements were determined according to the following standards [18]:

- Cu, Cr, Zn, Fe, Cd, Zn—PN-EN 14082: 2004 Food products—Determination of trace elements—Determination of lead, cadmium, zinc, copper, iron, and chromium by atomic absorption spectrometry (AAS) after dry mineralization [19].
- Ni—PN-A-86939-6:1998 Vegetable and animal oils and fats—Determination of heavy metal content by atomic emission spectrometry—Determination of nickel content [20].
- Mn—PN-EN ISO 6869:2002 Feed—Determination of calcium, copper, iron, magnesium, manganese, potassium, sodium, and zinc content—Atomic absorption spectrometry method [21].

### 2.8. Statistical Analysis

Data are presented as mean value and standard deviation (SD). The compliance of the distributions of quantitative variables with the theoretical normal distribution was checked using the Shapiro–Wilk test. The equality of variances was assessed using the Levene test. Depending on the results of the Shapiro–Wilk and Levene tests, the significance of differences between the mean values of quantitative parameters (place of residence, gender, age group) was assessed. The Student’s *t*-test for independent variables or the non-parametric Whitney U test was used. Statistical significance was set at *p* < 0.05. Statistical analyses were performed using Statistica 12 (TIBCO Software Inc., Palo Alto, CA, USA).

## 3. Results

### 3.1. Characteristics of Patients of Both Groups

The study was conducted from 2020 to 2021. The inclusion criteria were the clinical necessity of third molar extractions; a total of 28 patients were recruited from two regions in Lower Silesia, Poland (the industrial area of Glogow-Legnica) and a non-industrial area (Wroclaw). Specifically, 10 patients were from the industrial-mining region of Glogow-Legnica, and 18 were from the non-industrial area.

Despite the disparity in group sizes, no statistically significant age differences were observed between the groups (Table 1). The demographic characteristic that distinguished the populations was gender; in the non-industrial group, the majority of patients were women (38.3% vs. 16.7%, *p* = NS), but this difference was not statistically significant. Group classification was based on patients’ self-reported place of residence during interviews. The Legnica mining area group comprised patients who reported residing in this area for at least 18 years.

### 3.2. Content of Heavy Metals

Basic statistics of the content of toxic metals in the tested samples of patients from both groups are presented in Table 2, Table 3 and Table 4. Table 2 shows a comparison of the metal content based on the area inhabited by the patients. Table 3 presents a comparison based on gender, and Table 4 illustrates the level of heavy metals according to age.

The total heavy metal content, measured in µg/g, revealed significantly higher concentrations of copper and lead in patients from industrial areas compared to those from non-industrial regions (*p* = 0.017 and *p* = 0.048, respectively). Specifically, the copper levels in individuals residing in industrial areas were 0.310 ± 0.266, whereas in non-industrial areas, they were 0.122 ± 0.123. This indicates that residents of non-industrial areas had a 59.6% lower total copper content (Table 2). Similarly, the lead levels in patients from industrial areas were 0.552 ± 0.379, compared to 0.324 ± 0.203 from non-industrial areas, showing that residents of non-industrial areas had a 41.3% lower total lead content.

There was no statistically significant effect of patient gender on the content of manganese, chromium, nickel, copper, cadmium, lead, and zinc in teeth (*p* > 0.05). The only statistically significant difference was observed in the iron content (*p* = 0.0097), which was higher in men. The iron content values were 7.742 ± 6.770 for men and 3.116 ± 2.238 for women. This indicates that women had a 59.8% lower total iron content compared to men (Table 3).

There was no statistically significant effect of patients’ age on the content of manganese, chromium, nickel, iron, cadmium, lead, and zinc in teeth (*p* > 0.05). The only statistically significant difference was observed in the copper content (*p* = 0.047), which was higher in the 28–38 age group compared to the 18–27 age group. The copper content values were 0.310 ± 0.276 for the older patients and 0.141 ± 0.150 for the younger patients. This indicates that younger patients had a 54.5% lower total copper content in the 18–27 age group compared to the 28–38 age group (Table 4).

### 3.3. The Influence of the Living Environment on Isolated DSCs

The basic statistics for assessing the viability of cells isolated from the dental pulp in the examined samples of patients from both groups are presented in Table 5, Table 6 and Table 7. Table 5 shows a comparison of DSC (dental stem cell) cultures based on the patients’ places of residence. Table 6 shows the comparison by gender, and Table 7 shows the evaluation of cell cultures depending on the age of the patients.

**Figure 2:**
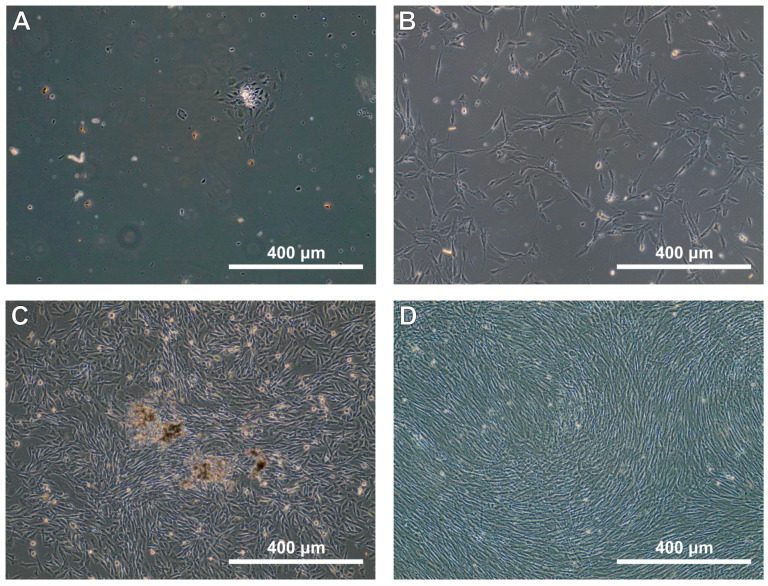
DSC cultures 48 h after isolation (**A**,**B**) and before the first passage (**C**,**D**). Cells isolated from an industrial district (**A**,**C**) from a non-industrial district (**B**,**D**).

Cell culture viability was statistically significantly lower in dental pulp samples from industrial areas. Specifically, the viability of cells from individuals residing in industrial areas was 84.410 ± 8.832, compared to 93.944 ± 5.208 in non-industrial areas (Table 5). Figure 2 shows an example culture of isolated dental stem cells (DSCs). In the case of cells isolated from pulp from industrial areas, we observe a few single cells after 48 h of incubation after isolation and many more from pulp from non-industrial areas. Similarly, the average culture doubling time was statistically significantly shorter in cultures of DSCs isolated from pulp from non-industrial areas, being more than twice as short: 41.2 ± 22.0 h in industrial areas versus 17.1 ± 4.8 h in non-industrial areas. The time to the first passage 24 h post-isolation was also statistically significantly shorter in the group of cell cultures isolated from non-industrial areas. Additionally, the number of cells after the first passage was statistically significantly higher in the group from non-industrial areas. The average number of cells after the first passage was 1.479 ± 0.250 million for tooth pulps from industrial areas (Figure 2B) and 1.746 ± 0.150 million for material from non-industrial areas (Figure 2D). This indicates that the efficiency of cell isolation from industrial areas is 15.3% lower compared to non-industrial areas (Table 5).

There was no statistically significant effect of patient age on any of the assessed culture parameters of cells isolated from dental pulp (*p* > 0.05) (Table 6).

Cell culture viability was statistically significantly lower in dental pulp samples from the 28–38 age group. Specifically, cell viability in individuals over 27 years of age was 85.750 ± 8.374, compared to 92.455 ± 7.254 in younger donors (Table 7). Similarly, the mean culture doubling time was statistically significantly shorter in DSC cultures isolated from the pulp of younger individuals, being 21.6 ± 11.5 h in donors under 27 years of age compared to 36.1 ± 26.0 h in older individuals. Additionally, the number of cells after the first passage was statistically significantly higher in the 18–27 age group. The average number of cells after the first passage was 1.709 ± 0.174 million for material from people under 27 years of age, compared to 1.506 ± 0.291 million for the pulp of teeth from people over 27 years of age. This indicates that the efficiency of cell isolation from older individuals is 11.9% lower than in younger donors (Table 7).

### 3.4. The Influence of DSCs Differentiation

The initial seeding density allowed for optimal cell growth and confluence. The use of pre-coated plates with MSC Attachment Solution facilitated cell adhesion and uniform growth. Transitioning to an osteogenic differentiation medium at approximately 80% confluence ensured a robust initiation of the differentiation process. Extended incubation times were correlated with increased mineralization, as evidenced by more intense Alizarin Red S (ARS) staining.

**Figure 3:**
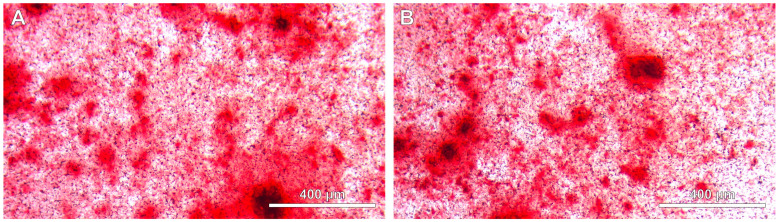
DSC cultures after differentiation in osteogenic medium: (**A**) patient from the industrial district, (**B**) patient from the non-industrial district.

Cells incubated for longer periods (up to 21 days) in the osteogenic medium demonstrated higher levels of mineralization. The periodic replacement of the differentiation medium every 2 to 3 days was crucial for maintaining the differentiation process and providing necessary nutrients. The ARS staining provided clear evidence of mineral deposition. The intensity of the ARS staining was directly proportional to the incubation time, confirming successful osteogenic differentiation. Quantitative analysis of the ARS staining can further elucidate the extent of mineralization. Importantly, there were no statistically significant differences in the level of differentiation and mineralization (Figure 3) regardless of the origin of the explant—whether the tooth pulp came from a person living in an industrial area or not and whether it came from a woman or a man.

## 4. Discussion

Stem cells isolated from dental pulp (DPSCs) show significant potential in regenerative medicine. Currently, research is being conducted on their application in treating conditions such as neurodegenerative diseases, spinal cord injuries, and heart diseases [22]. DPSCs have a high capacity for differentiating into various cell types, making them a promising tool for regenerating damaged tissues. The application of DPSCs in the regeneration of dental pulp and dentin tissue has direct significance for aesthetic dentistry, especially in the context of preserving and restoring teeth and periodontal tissue, which is important for the aesthetics of the smile and oral health [23]. The potential clinical applications of DPSCs, including their ability to regenerate bone, are significant in the context of reconstructing bone structures in the craniofacial area, and thus for facial aesthetics [24]. Research is being conducted into using lasers to help grow new sets of teeth, e.g., by using lasers to activate stem cells, researchers were able to stimulate tooth growth in rats [25]. Future research focuses on improving the efficiency of differentiating these cells and developing new methods for delivering them to injury sites. Understanding the tissue microenvironment and immune mechanisms is crucial for enhancing the effectiveness of therapies using DPSCs [22].

The isolation of DPSCs is quite efficient; after a few passages, several million cells can be obtained from a small amount of pulp. The undeniable advantage of this method of obtaining stem cells is the minimally invasive nature of the material collection, which is medically indicated, in contrast to cells obtained from bone marrow or adipose tissue [15].

As indicated by the team led by Juli Baronova, the problem of reconstructing teeth from isolated stem cells from dental pulp is quite complex and costly. There is still a lack of developed materials for guiding regeneration in the tissue engineering process and for releasing drugs to prevent, among other things, inflammatory conditions [15].

In recent studies, the utilization of autogenous dentin has been proposed as an innovative alternative for preserving alveolar bone post-extraction. This method has shown promising results in maintaining bone volume and quality, offering a viable option for bone regeneration [26,27].

We decided to investigate whether, in today’s times, with such a developed economy but significant environmental pollution, this has an impact on the quality and quantity of cells obtained from dental pulp. The material collected came from industrial areas of Lower Silesia in Poland and the Wroclaw agglomeration.

Wychowanski and Malkiewicz analyzed the occurrence of trace elements in the enamel and dentin of impacted third molars in patients from urban and agricultural areas of Masovia, Poland [28]. They collected dental material from 30 patients aged 26–37. Their research indicates that the enamel and dentin of individuals living in cities contain significantly higher levels of lead and cadmium compared to those living in agricultural areas, as well as elevated levels of manganese and chromium [28]. Similarly, Bryła and co-authors noted an increased cadmium content in the dental tissues of residents of the Wroclaw agglomeration, which increased with age but was independent of gender [29]. In our research, we observed statistically significantly higher levels of copper and lead deposited in teeth from industrial areas. Additionally, similar to Wychowanski’s findings, higher but not statistically significant levels of manganese, chromium, and zinc were observed in the urban agglomeration compared to the Glogow-Legnica district. Consequently, this affected the viability of cell cultures immediately after isolation. A statistically significant difference was observed: in non-industrial areas, the viability level was above 90%, while in industrial areas, it was around 84%. Cells of patients from non-industrial areas proliferated significantly faster, and in the first passage, about 300,000 more cells were obtained. At the same time, the yield of cells was sufficient in both cases for potential transfer to scaffolds. An important aspect is the demonstration that in clinical practice, where tooth extractions are often performed after the working hours of cell culture laboratories, especially in emergency cases, storing the pulp for 24–48 hours does not affect the viability of isolated cells. This is of great importance from the perspective of the patient and the dentist in the potential future use of tissue engineering. Most importantly, this study showed that regardless of the source of tooth pulp for isolating DPSCs, there was no effect on osteogenic differentiation.

In this study, no significant differences were observed in the content of heavy metal elements between genders, except of iron levels. Our findings diverge from those reported by Bryła et al. [29]. In our group, we identified a statistically significant higher iron content in samples obtained from men. This phenomenon can be attributed to the fact that iron is a microelement present in bones that accumulates with age, and its concentration is primarily dependent on serum levels. In conditions of iron excess, the hepatic protein hepcidin inhibits the absorption of iron from erythrocytes into the bloodstream and reduces the release of iron from macrophages. Maciejewska reported similar findings, highlighting the impact of other microelements on bone development [30]. Furthermore, WHO guidelines on iron supplementation recommend that women of reproductive age, particularly those who menstruate, should take iron prophylactically to prevent deficiencies. Research indicates that iron supplementation in menstruating women enhances hemoglobin levels and mitigates the risk of anemia [31]. Consequently, despite supplementation, physiological processes such as menstruation, pregnancy, childbirth, and lactation result in lower iron deposition in bones in women compared to men. However, this difference did not affect the effectiveness of isolated DPSCs.

In our study, we observed a statistically significant increase in copper content in the bones of teeth in the 28–38 years age group compared to the 18–27 years group. These results are consistent with the findings of Fischer and colleagues, who noted an increase in copper and other elements in the permanent teeth of children aged 5–14 years [32]. On the other hand, Bryła and colleagues did not observe differences in copper content regardless of age [29]. This discrepancy may be because Fischer’s study included children up to 14 years old, who are in a phase of continuous development and have been exposed to environmental factors for a shorter period. After the complete saturation of the hard tissues of the tooth with minerals, which can take several years after the tooth has rooted, this level remains constant throughout life. In our study, we analyzed only third molars, which erupt late, usually between the ages of 17 and 24, although this time can vary individually [33]. Therefore, the saturation of elements in these teeth may be prolonged, explaining the differences. However, copper content does not seem to affect the quality and quantity of isolated DSCs. Differences in the yield of isolated DSCs depending on the age of the patient may be because stem cells in older individuals tend to divide more slowly and have a reduced capacity for proliferation. This may be caused by changes in signaling pathways, such as the mTOR pathway, which regulates cell growth and the cell cycle. Stem cells in older individuals may have altered metabolism, affecting their ability to divide and differentiate, leading to a reduced number of stem cells available for isolation [34,35].

Limitations: While this study offers important insights into the effects of environmental pollution on DSC viability and proliferation, several limitations should be acknowledged. First, the relatively small sample size (28 patients) limits the generalizability of the findings. The statistical power of the study was estimated at 49.7%, which is moderate but reflects a typical range for preliminary investigations. This level of power still enabled the detection of statistically significant differences between the groups, but a larger sample size would enhance the robustness and reliability of the conclusions.

Moreover, the classification of patients based solely on self-reported place of residence without controlling for dietary habits, smoking, or occupational exposure could introduce bias. These factors are known to affect heavy metal accumulation and may have contributed to variability in DSC viability. Future studies with larger cohorts and more controlled variables are necessary to confirm these findings and further explore the environmental impact on DSC proliferation. Additionally, this study’s cross-sectional design limits our ability to observe changes in heavy metal content and DSC viability over time. Future longitudinal studies are planned to track these changes and offer a more comprehensive understanding of how prolonged exposure to environmental pollutants impacts DSC characteristics.

## 5. Conclusions

This study highlights the significant impact of environmental pollution on the quality and viability of dental stem cells (DSCs) extracted from impacted third molars. By comparing DSCs from patients from industrial and non-industrial areas, the research reveals the adverse effects of heavy metals on oral health and stem cell proliferation. Findings show higher copper and lead levels in dental tissues from patients in industrial areas, correlating with lower cell culture viability and slower proliferation. These results suggest that pollutants compromise the regenerative potential of DSCs. While these findings provide valuable insights into the impact of environmental pollution on the viability and proliferation of DSCs, it is important to note that this study represents preliminary research.

Our primary goal is to develop a reproducible and scalable methodology for isolating and utilizing DSCs in clinical practice. The ultimate objective is to register a DSC-based product with the European Medicines Agency (EMA) as an Advanced Therapy Medicinal Product (ATMP) and establish a reliable production process. This would enable the use of DSCs in regenerative treatments, particularly for patients exposed to environmental pollutants, offering a personalized approach to regenerative medicine.

The concentration of essential elements and activity of dental pulp stem cells are crucial for effective bone regeneration in the maxillofacial region. Sufficient levels of calcium and phosphate support mineralization, while stem cell activity enhances tissue formation, improving graft stability and durability.

The study also emphasizes the need to consider environmental factors in dental medicine. While no significant gender differences in heavy metal content were found, except elevated iron levels in men, age-related variations in copper were observed. These findings are key for developing targeted interventions for populations exposed to pollution. Overall, this research emphasizes the need for stricter environmental regulations and public health policies to reduce exposure to harmful pollutants. Further studies are needed to explore mitigation strategies and enhance clinical outcomes for individuals affected by environmental pollution.

## Figures and Tables

**Table 1:** Characteristics of patients of both groups.

Feature	Industrial District (n = 10)		Non-Industrial District (n = 18)		All		*p*-Value
	n	%	n	%	n	%	
Sex							
Women	6	60.0	15	83.3	21	75.0	0.327
Men	4	40.0	3	16.7	7	25.0	
Age group							
18–27 years	7	70.0	14	77.8	21	75.0	0.757
28–38 years	3	30.0	4	22.2	7	25.0	
Age (years)							
Mean ± SD	24.0 ± 5.9		26.0 ± 4.4		25.0 ± 5.0		0.300

**Table 2:** Content of heavy metals in teeth from people from industrial and non-industrial areas.

Heavy Metals [µg/g]	Industrial District (Mean ± SD)	Non-Industrial District (Mean ± SD)	*p*-Value
Mn	0.164 ± 0.090	0.239 ± 0.229	0.331
Cr	0.000 ± 0.000	0.088 ± 0.372	0.446
Ni	0.000 ± 0.000	0.000 ± 0.000	1.000
Cu	0.310 ± 0.266	0.122 ± 0.125	0.017
Fe	4.899 ± 2.775	3.925 ± 4.924	0.571
Cd	0.064 ± 0.033	0.062 ± 0.021	0.814
Pb	0.551 ± 0.379	0.324 ± 0.203	0.048
Zn	86.300 ± 29.104	103.243 ± 30.305	0.163

**Table 3:** Content of heavy metals in teeth taken from patients of different sexes.

Heavy Metals [µg/g]	Women	Men	*p*-Value
Mn	0.221 ± 0.216	0.185 ± 0.099	0.676
Cr	0.000 ± 0.000	0.225 ± 0.596	0.083
Ni	0.000 ± 0.000	0.000 ± 0.000	1.000
Cu	0.183 ± 0.168	0.210 ± 0.307	0.765
Fe	3.116 ± 2.238	7.742 ± 6.770	0.0097
Cd	0.064 ± 0.026	0.058 ± 0.026	0.572
Pb	0.405 ± 0.314	0.406 ± 0.242	0.992
Zn	100.576 ± 30.177	87.041 ± 31.377	0.318

**Table 4:** Content of heavy metals in teeth taken from patients of different ages.

Heavy Metals [µg/g]	18–27	28–38	*p*-Value
Mn	0.176 ± 0.091	0.302 ± 0.328	0.121
Cr	0.079 ± 0.353	0.000 ± 0.000	0.537
Ni	0.000 ± 0.000	0.000 ± 0.000	1.000
Cu	0.141 ± 0.150	0.310 ± 0.278	0.047
Fe	4.783 ± 4.728	2.997 ± 2.523	0.324
Cd	0.063 ± 0.024	0.061 ± 0.032	0.870
Pb	0.372 ± 0.306	0.487 ± 0.260	0.362
Zn	93.534 ± 26.609	106.337 ± 39.084	0.325

**Table 5:** Evaluation of DSC cultures from human teeth from industrial and non-industrial areas.

Parameters	Industrial District (Mean ± SD)	Non-Industrial District (Mean ± SD)	*p*-Value
Viability cells	84.410 ± 8.832	93.944 ± 5.208	0.001
Number of cells obtained (million) in the first passage	1.479 ± 0.250	1.746 ± 0.150	0.002
24 h after isolation and the first passage	9.3 ± 2.6	5.7 ± 0.8	<0.001
Average doubling time (h)	41.2 ± 22.0	17.1 ± 4.8	<0.001

**Table 6:** Evaluation of DSC cultures from human teeth from patients of different sexes.

Parameters	Women	Men	*p*-Value
Viability cells	91.033 ± 7.424	89.057 ± 10.204	0.583
Number of cells obtained (million) in the first passage	1.657 ± 0.202	1.632 ± 0.312	0.802
24 h after isolation and the first passage	6.7 ± 2.1	7.9 ± 3.2	0.261
Average doubling time (h)	23.5 ± 14.5	32.3 ± 25.3	0.307

**Table 7:** Evaluation of DSC cultures from human teeth from patients of different ages.

Parameters	18–27 Years	28–38 Years	*p*-Value
Viability cells	92.455 ± 7.254	85.750 ± 8.374	0.044
Number of cells obtained (million) in the first passage	1.709 ± 0.174	1.506 ± 0.291	0.031
24 h after isolation and the first passage	6.5 ± 1.9	8.1 ± 3.2	0.108
Average doubling time (h)	21.6 ± 11.5	36.1 ± 26.0	0.048

## Data Availability

The original contributions presented in the study are included in the Supplementary Material; further inquiries can be directed to the corresponding author.

## Supplementary Materials

The following supporting information can be downloaded at: https://www.mdpi.com/article/10.3390/jcm13185465/s1.
